# Household factors associated with access to insecticide-treated nets and house modification in Bagamoyo and Ulanga districts, Tanzania

**DOI:** 10.1186/s12936-020-03303-8

**Published:** 2020-06-23

**Authors:** Olukayode G. Odufuwa, Amanda Ross, Yeromin P. Mlacha, Omary Juma, Selemani Mmbaga, Daniel Msellemu, Sarah Moore

**Affiliations:** 1 Ifakara Health Institute, Bagamoyo, Tanzania; 2 Swiss Tropical and Public Health Institute, Basel, Switzerland; 3 University of Basel, Basel, Switzerland

**Keywords:** Access, Insecticide Treated nets, ITNs, House modification, Malaria, Demographic, Socioeconomic, Vector-borne diseases, Tanzania

## Abstract

**Background:**

Insecticide-treated nets (ITNs) and house modifications are proven vector control tools, yet in most regions, full coverage has not been achieved. This study investigates household factors associated with access to ITNs and house modification in Tanzania.

**Methods:**

Baseline cross-sectional survey data from previous studies on spatial repellants and indoor residual spray evaluation was analysed from 6757 households in Bagamoyo (60 km north of Dar es Salaam) and 1241 households in Ulanga (a remote rural area in southeast Tanzania), respectively. Regression models were used to estimate the associations between the outcomes: population access to ITNs, access to ITN per sleeping spaces, window screens and closed eaves, and the covariates household size, age, gender, pregnancy, education, house size, house modification (window screens and closed eaves) and wealth.

**Results:**

Population access to ITNs (households with one ITN per two people that stayed in the house the previous night of the survey) was 69% (n = 4663) and access to ITNs per sleeping spaces (households with enough ITNs to cover all sleeping spaces used the previous night of the survey) was 45% (n = 3010) in Bagamoyo, 3 years after the last mass campaign. These findings are both lower than the least 80% coverage target of the Tanzania National Malaria Strategic Plan (Tanzania NMSP). In Ulanga, population access to ITNs was 92% (n = 1143) and ITNs per sleeping spaces was 88% (n = 1093), 1 year after the last Universal Coverage Campaign (UCC). Increased household size was significantly associated with lower access to ITNs even shortly after UCC. House modification was common in both areas but influenced by wealth. In Bagamoyo, screened windows were more common than closed eaves (65% *vs* 13%), whereas in Ulanga more houses had closed eaves than window screens (55% *vs* 12%).

**Conclusion:**

Population access to ITNs was substantially lower than the targets of the Tanzania NMSP after 3 years and lower among larger households after 1 year following ITN campaign. House modification was common in both areas, associated with wealth. Improved access to ITNs and window screens through subsidies and Behaviour Change Communication (BCC) strategies, especially among large and poor households and those headed by people with a low level of education, could maximize the uptake of a combination of these two interventions.

## Background

Insecticide-treated nets (ITNs) have made the greatest contribution to the reduction in malaria burden in sub-Saharan Africa [1], through both individual and community effects [2]. The community effect works better when there is a high coverage of ITNs [2]. Hence, ITNs are recommended by the World Health Organization (WHO) to be universally and continuously distributed to all people at risk in malaria endemic regions [3]. Multiple ITN delivery strategies and campaigns have been implemented across sub-Saharan Africa, either targeting specific malaria risk populations (children under 5 years of age and pregnant women), or targeting the entire population through health facilities, antenatal clinics (ANC), schools, markets, door-to-door and other methods [4–7]. Despite more than a decade of ITN campaigns, an average of about 60% of the population in malaria endemic areas, still do not have access to an ITN [8], indicating that greater malaria control could be attained with improved access to this highly effective intervention.

**Fig. 1:**
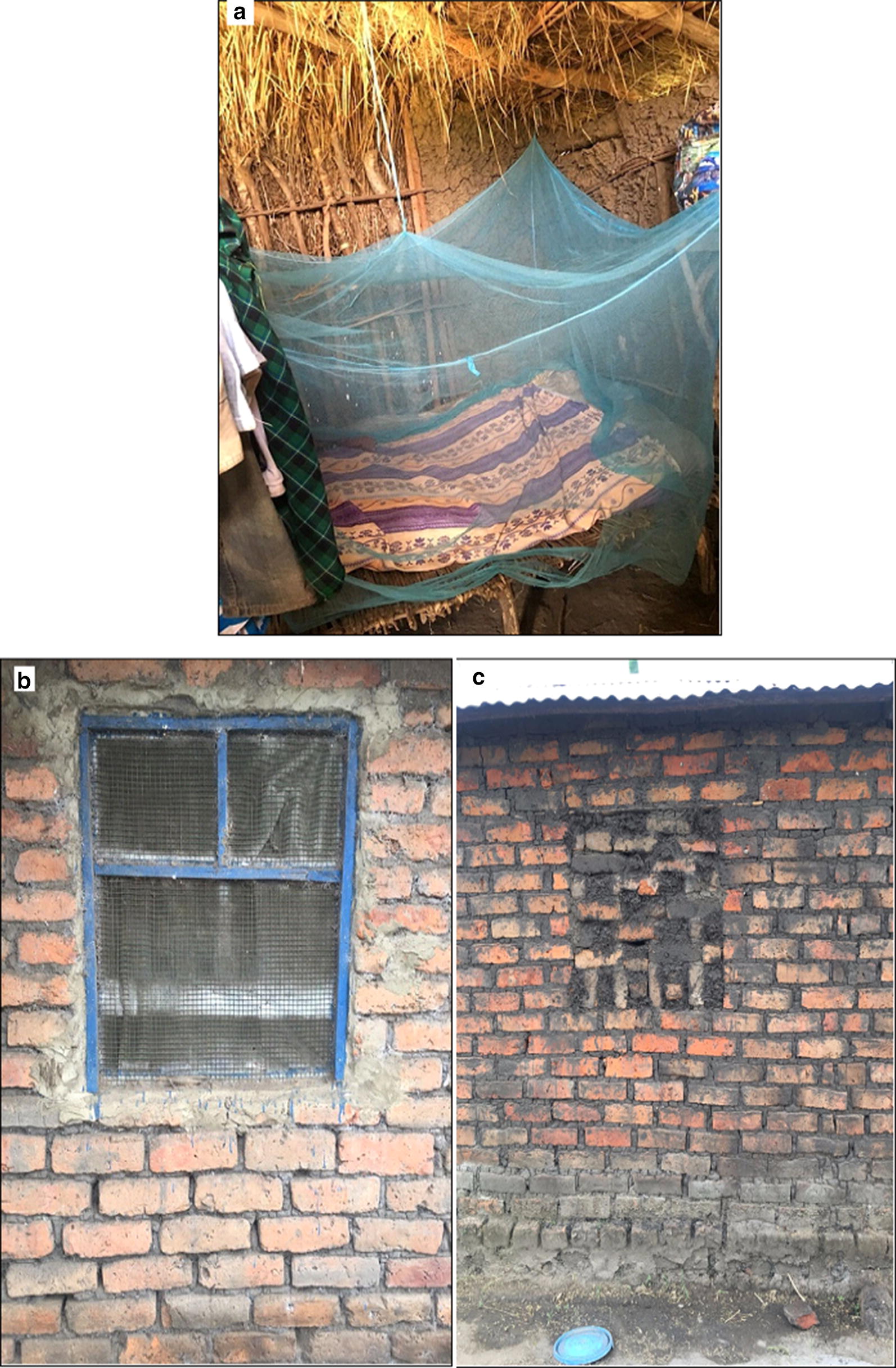
**a** An ITN hanging in a rural Tanzanian home. **b** Screened window. **c** Eaves and ventilation bricks closed with mud to prevent mosquito entry

House modification provides additional protection from all mosquitoes when people are indoors but not under their ITN [9], The installation of window screens or blocking of eaves (Fig. 1) acts to create barrier against mosquito entry and have been shown to be associated with lower malaria infection [10] and are a control tool for other vector-borne diseases such as dengue [11], arbovirus and lymphatic filariasis [12]. They (1) require no active compliance, (2) tend to be long-lasting and (3) protect all members of a household, 4) improving house ventilation and (5) preventing mosquito entry and nuisance [13, 14]. House modification is not currently delivered operationally as a vector control tool. It is facing limited support from government or other organizations [15], being generally considered too costly to install or difficult to implement [14] as a public health intervention. However, there is evidence of an increasing number of houses with window screens and closed eaves among the urban Tanzanian population especially window screens [13].

This paper reports the household factors associated with access to ITNs and house modification between two rural populations: one far from and one close to an urban centre, as it is known that access to economic centers affects the economic, education, and health status of populations [16]. This work will provide information to policy-makers on identifying the gaps among different household settings and maximizing access to and use of these existing malaria control tools.

## Methods

### Study area and socio-demographic characteristics

**Fig. 2:**
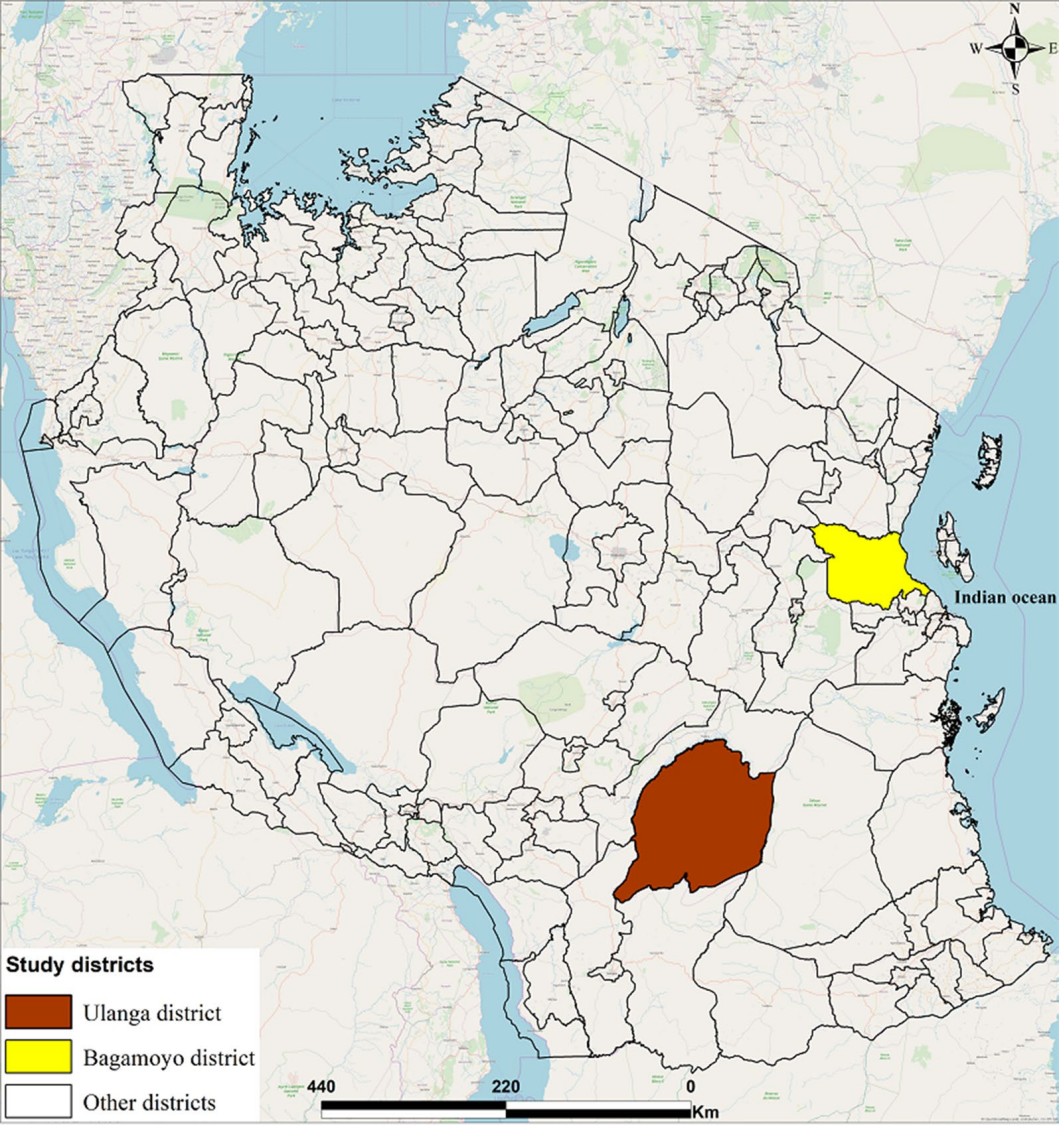
Geographical location of Bagamoyo and Ulanga districts in Tanzania. Openstreet base map obtained from the ArcGIS plugin

The study was conducted in Bagamoyo and Ulanga districts (Fig. 2). Bagamoyo district is located on the east coast of Tanzania, approximately 60 km north of Dar es Salaam, the economic hub of the country [17] and Ulanga district is located in rural south-east Tanzania, 300 km from the regional city of Morogoro and 500 km from Dar es Salaam [18]. In Bagamoyo and Ulanga, the average household size was 4.4 and 4.9 persons per household, respectively according to the 2012 Tanzania National Census [19]. Adult literacy in Bagamoyo was 58% and 66% in Ulanga [20]. The average rainfall and temperature are 1200 to 2100 mm per year and approximately 28 °C in both districts, with slightly higher average rainfall per year and temperature in Bagamoyo due to its coastal location. Above 70% of the residents in both districts own pieces of land and the majority of them engage in subsistence farming [18, 21]. The pattern of climatic conditions and land use are contributing factors toward malaria transmission [22].

### Study design

The study is a secondary analysis of two baseline cross-sectional surveys from a trial on spatial repellents in Bagamoyo [23], and an insecticide residual spray (IRS) evaluation in Ulanga [24]. The surveys were continuously conducted randomly in nine villages in the Bagamoyo district from November 2014 to October 2015 and eight villages in Ulanga district from December 2016 to June 2017, covering both dry and rainy season.

### Household survey data collection

Data were collected using a structured questionnaire upon written informed consent from an adult household member. The questionnaire surveyors collected information on ITN coverage indicators: (1) households with at least one ITN for every two people that slept in the household [25] and (2) households with one ITN per sleeping space [26], to investigate the coverage achieved by the Universal Coverage Campaign (UCC) conducted from the mid-year 2015 to 2017, to cover all sleeping spaces that were recorded to be unreached [4].

Information was collected on demographic and socio-economic characteristics, household geographical coordinates (longitude, latitude and elevation) and notes on the house structure were made based on observation by the interviewers. Information on age, gender and level of formal education was collected for the head of households, and the total number of pregnant women within each household was also recorded. The surveys were delivered using similar questionnaires in the two sites except that in Ulanga, data on age, gender and educational status of heads of households and the geographical coordinates were not collected.

### Data management and analysis

The paper questionnaires were double-entered using EpiData software [27] and analysis was carried out using STATA 14.2 [28]. The socioeconomic status of each household was derived from a weighted score using principal component analysis (PCA), categorized into quintiles: lowest, low, middle, high and highest [29]. PCA analysis was conducted separately in Bagamoyo and Ulanga. In Bagamoyo, the variables selected for the PCA analysis were ownership of durable asset (radio, mobile phone, fan, television, iron, refrigerator and cable television), farm animals (chickens, ducks, goats, cows, dogs), a means of transportation (animal cart, bicycle, motorcycle, canoe, tricycle, ark, and car), access to utilities (water and toilet) and a light source (candle, electricity, battery/solar panel, hurricane lamp, kerosene and fire), and house structure (wall, roof, ceiling and floor). Similar variables were selected for the PCA analysis in Ulanga except that torch-light was added as a form of light source, and, other variables such as fan, iron, refrigerator, animal cart, canoe, tricycle, ark and car were excluded, as none of the households in Ulanga possessed them.

The population access to ITNs was defined as the whether or not there was at least one net available on the previous night per two people who slept in the household on the previous night. Access to ITN per sleeping space was defined as available net the previous night in each sleeping space which had been used the previous night. Indicators of population access to ITNs and access to ITNs for sleeping spaces were defined in line with the household survey indicators for malaria control [30].

The outcomes, population access to ITNs, access to ITN per sleeping spaces, presence of window screens and the presence of closed eaves were analysed using a logistic regression model. The associations between the outcomes and the covariates of household size, age, gender, pregnancy, education, house size, house modification (window screens and closed eaves), and socioeconomic quintiles were estimated. The village was included in the regression model as a random effect to account for clustering. The ages of the heads of households were categorized into three levels: young adults “18–24”, adult “25–49” and old adults “50–99”. Household size was also categorized into two levels, small and average household size “1–5” and large household size “6 and above”. The number of doors and windows were added together and categorized into two levels: small and average house size “1–4” and large house size “5 and above” to indicate the physical size of houses.

All covariates and interaction between education and wealth status were considered in the analysis based on findings from past literature [31–35]. The likelihood-ratio test was conducted by comparing the model with all covariates against the model without each covariate one after the other and the p-values were reported. Collinearity was investigated using the Collin program in STATA and no unacceptable correlation was suspected [36].

## Results

### Demographic and socioeconomic characteristics of households

A total of 6757 households in Bagamoyo and 1241 in Ulanga districts were surveyed (Table 1). The mean household size in Bagamoyo and Ulanga districts was 4.38 and 3.78, respectively. The mean number of sleeping spaces in Bagamoyo was 2.67 and 1.85 in Ulanga. From data collected in Bagamoyo only, fewer households (9% [n = 594]) were headed by young adults (18 to 24 years). Roughly, 77% [n = 5174] of the households in Bagamoyo were headed by a male and most of the head of households had primary education (71% [n = 4829]). The proportion of large houses was greater in Bagamoyo (44% [n = 2999]) than Ulanga (26% [n = 327]), indicating more wealthy households in Bagamoyo than Ulanga district (Table 1).

**Table 1:** The number and percentage distribution of household factors, population access to ITN, access to ITN for all sleeping spaces, window screens and closed eaves in Bagamoyo and Ulanga district

Districts	Bagamoyo	Ulanga	Bagamoyo	Ulanga	Bagamoyo	Ulanga	Bagamoyo	Ulanga	Bagamoyo	Ulanga
Variables	Household Characteristics n (%)		Population access to ITNs for all sleeper n (%)		Access to ITNs for all sleeping spaces n (%)		Houses with window screens n (%)		Houses with closed eaves n (%)	
Total (N)	6757	1241	4663 (69)	1143 (92)	3010 (44)	1093 (88)	4392 (65)	150 (12)	904 (13)	679 (55)
Household size										
1 to 5	4809 (71)	972 (78)	3418 (71)	925 (95)	2356 (49)	866 (89)	3188 (66)	102 (11)	664 (14)	551 (57)
6 to 20	1948 (29)	269 (22)	1245 (64)	218 (81)	654 (34)	227 (84)	1204 (62)	48 (18)	240 (12)	128 (48)
Age group of HH head										
18–24	594 (9)	–	388 (65)	–	296 (50)	–	436 (73)	–	82 (14)	–
25–49	3936 (58)	–	2756 (70)	–	1826 (46)	–	2761 (70)	–	596 (15)	–
50– 99	2227 (33)	–	1519 (68)	–	888 (40)	–	1195 (54)	–	226 (10)	–
Gender of HH head										
Male	5172 (77)	–	3543 (69)	–	2286 (44)	–	3402 (66)	–	718 (14)	–
Female	1585 (24)	–	1120 (71)	–	724 (46)	–	990 (63)	–	186 (12)	–
HH with Pregnant women										
No	6461 (96)	1193 (96)	4463 (69)	1106 (93)	2890 (45)	1056 (89)	4182 (65)	141 (12)	872 (14)	656 (55)
Yes	296 (4)	48 (4)	200 (68)	37 (77)	120 (41)	37 (77)	210 (71)	9 (19)	32 (11)	23 (48)
Education level of HH head										
None	1224 (18)	–	772 (63)	–	447 (37)	–	577 (47)	–	69 (6)	–
Primary	4829 (71)	–	3344 (69)	–	2161 (45)	–	3244 (67)	–	599 (12)	–
Secondary and Above	704 (10)	–	547 (78)	–	402 (57)	–	571 (81)	–	236 (34)	–
House size										
Small and average	3758 (56)	913 (74)	2431 (65)	837 (92)	1725 (46)	820 (90)	2210 (59)	61 (7)	416 (11)	545 (60)
Large	2999 (44)	327 (26)	2232 (74)	305 (93)	1285 (43)	272 (83)	2182 (73)	89 (27)	488 (16)	133 (41)
House modification										
None	2333 (33)	482 (39)	1414 (63)	431 (89)	855 (38)	416 (86)	–	–	–	–
Closed eaves	132 (2)	609 (49)	92 (70)	570 (94)	69 (52)	546 (90)	–	–	–	–
Window screens	3620 (54)	80 (7)	2528 (70)	74 (93)	1631 (45)	72 (90)	–	–	–	–
Screened windows and eaves	772 (11)	70 (6)	629 (82)	68 (97)	455 (59)	59 (84)	–	–	–	–
Wealth Quintile										
Lowest	1346 (20)	215 (17)	856 (64)	211 (98)	513 (38)	198 (92)	536 (40)	2 (1)	47 (4)	121 (56)
Low	1345 (20)	278 (22)	871 (65)	246 (89)	555 (41)	245 (88)	797 (59)	12 (4)	72 (5)	178 (64)
Middle	1363 (20)	243 (20)	926 (68)	220 (91)	594 (44)	204 (84)	965 (71)	21 (9)	77 (6)	139 (57)
High	1321 (20)	250 (20)	893 (68)	227 (91)	554 (42)	220 (88)	909 (69)	28 (11)	100 (8)	118 (47)
Highest	1382 (21)	255 (21)	1117 (81)	239 (94)	794 (58)	226 (89)	1185 (86)	87 (34)	608 (44)	123 (48)

### Access to ITNs in the population

In Bagamoyo, 3 years after a mass distribution campaign, population access to ITNs (proportion of households with one ITN for two potential users that stayed in the house the previous night of the survey) was estimated as 69% [n = 4663]. Access to one ITN per sleeping space (total number of households with enough ITNs to cover all sleeping spaces used the previous night of the survey) was estimated as 45% [n = 3010]. These estimates are lower than the least 80% coverage targets of the Tanzania National Malaria Operational Plan 2019 [37]. In Ulanga, 1 year after a mass distribution campaign, population access to ITN was 92% [n = 1143] and ITN per sleeping spaces was 88% [n = 1093].

### Household factors associated with access to ITNs in Bagamoyo and Ulanga

The household factors associated with having greater access to ITNs for two potential sleepers in Bagamoyo in the multivariable analysis were having screened windows or closed eaves, a smaller household size, older head of household, a higher level of education, and a larger physical house size. In Ulanga, a smaller household size and a larger physical house size were both also significantly associated with having access to ITN for two potential sleepers. Wealth quintile was also associated, but whereas in Bagamoyo the higher quintile had the highest access, in Ulanga it was the lowest wealth quintile (Table 2).

**Table 2:** Association between household factors and having access to ITNs for two potential sleepers in the population

Districts	Bagamoyo					Ulanga				
Models	Univariate		Multivariable			Univariate		Multivariable		
Co-variates	OR	95% CI	OR	95% CI	P-value	OR	95% CI	OR	95% CI	P-value
Household size										
1–5	1		1		0.001	1		1		0.001
6 and above	0.72	0.64–0.81	0.63	0.55–0.71		0.22	0.14–0.33	0.31	0.18–0.52	
Age group of HH head										
18–24	1		1		0.041	–	–	–	–	–
25–49	1.24	1.03–1.49	1.26	1.04–1.53		–	–	–	–	–
50 and above	1.14	0.94–1.38	1.16	0.94–1.43		–	–	–	–	–
Gender of HH head										
Male	1		1		0.101	–	–	–	–	–
Female	1.11	0.98–1.25	1.12	0.98–1.28		–	–	–	–	–
Households with pregnant women										
No	1		1		0.910	1		1		0.170
Yes	0.93	0.73–1.20	1.02	0.78–1.32		0.26	0.13 - 0.54	0.51	0.19 - 1.32	
Level of formal Education of HH head										
None	1		1		0.001	–	–	–	–	–
Primary	1.32	1.16–1.50	1.31	1.13–1.52		–	–	–	–	–
Secondary and above	2.04	1.65–2.52	1.55	1.22–1.96		–	–	–	–	–
House size										
Small and average	1		1		0.001	1		1		0.010
Large	1.59	1.71–1.96	1.58	1.39–1.78		1.26	0.77–2.06	2.34	1.20–4.58	
House entry protected										
None	1		1		0.001	1		1		0.063
Closed eaves	1.33	0.91–1.95	0.95	0.64–1.43		1.73	1.12–2.67	1.72	1.00–2.93	
Screened windows	1.34	1.20–1.50	1.27	1.12–1.44		1.46	0.60–3.52	1.75	0.62–4.95	
Screened windows and closed eaves	2.55	2.08–3.11	1.53	1.21–1.94		4.02	0.96–16.91	4.41	0.93–20.90	
Wealth Quintile										
Lowest	1		1		0.001	1		1		0.041
Low	1.05	0.90–1.23	0.86	0.73–1.02		0.15	0.05–0.42	0.22	0.07–0.67	
Middle	1.21	1.03–1.42	1.01	0.85–1.20		0.18	0.06–0.53	0.27	0.09–0.86	
High	1.19	1.02–1.40	0.87	0.73–1.03		0.19	0.06–0.55	0.24	0.08–0.77	
Highest	2.41	2.03–2.87	1.41	1.15–1.73		0.28	0.09–0.86	0.22	0.06–0.76	

The first level in each variable with the value 1 represents the reference group

Similar variables were associated with access to ITNs per sleeping space (Table 3) in Bagamoyo and Ulanga, except that in Ulanga there was only evidence for greater access to ITN by sleeping space in houses that were smaller in physical size.

**Table 3:** Association between household factors and having access to ITNs for all sleeping spaces

Districts	Bagamoyo					Ulanga				
Models	Univariate		Multivariable			Univariate		Multivariable		
Covariates	OR	95% CI	OR	95% CI	P-value	OR	95% CI	OR	95% CI	P-value
Household size										
1 to 5	1		1		0.001	1		1		0.78
6 to 20	0.53	0.47–0.59	0.58	0.51–0.66		0.66	0.45–0.97	1.07	0.68–1.66	
Age group of HH head										
18–24	1		1		0.006	–	–	–	–	–
25–49	0.87	0.73–1.04	1.01	0.84–1.22		–	–	–	–	–
50–99	0.67	0.56–0.80	0.83	0.68–1.02		–	–	–	–	–
Gender of HH head										
Male	1		1		0.234	–	–	–	–	–
Female	1.06	0.95–1.19	1.08	0.95–1.22		–	–	–	–	–
Households with pregnant women										
No	1		1		0.357	1		1		0.15
Yes	0.84	0.66–1.07	0.89	0.69–1.14		0.44	0.22–0.86	0.56	0.26–1.20	
Level of formal education of HH head										
None	1		1		0.001	–	–	–	–	–
Primary	1.41	1.24–1.60	1.41	1.22–1.63		–	–	–	–	–
Secondary and above	2.31	1.91–2.80	1.75	1.41–2.18		–	–	–	–	–
House size										
Small and average	1		1		0.002	1		1		0.002
Large	0.88	0.80–0.97	0.83	0.74–0.93		0.56	0.39–0.80	0.50	0.32–0.78	
House entry protected										
None	1		1		0.001	1		1		0.583
Closed eaves	1.77	1.24–2.51	1.27	0.88–1.85		1.38	0.95–1.99	1.17	0.79–1.73	
Screened windows	1.32	1.19–1.47	1.35	1.20–1.53		1.43	0.66–3.10	1.65	0.72–3.81	
Screened windows and closed eaves	2.31	1.96–2.73	1.54	1.26–1.89		0.85	0.43 - 1.70	0.96	0.45 - 2.05	
Wealth Quintile										
Lowest	1		1		0.001	1		1		0.231
Low	1.14	0.98 - 1.33	0.94	0.80 - 1.11		0.64	0.34 - 1.18	0.67	0.35 - 1.27	
Middle	1.25	1.08–1.46	1.13	0.96–1.34		0.45	0.25–0.82	0.54	0.28–1.02	
High	1.17	1.00–1.37	0.94	0.79–1.11		0.63	0.34–1.18	0.82	0.42–1.61	
Highest	2.19	1.88–2.56	1.52	1.26–1.82		0.67	0.36–1.25	0.9	0.43–1.89	

The first level in each variable with the value 1 represents the reference group

### House modifications (Houses having window screens and closed eaves) in the population

In Bagamoyo, 65% [n = 4392] of houses had window screens, compared to 12% [n = 150] in Ulanga. Closed eaves were observed in 13% [n = 904] of houses in Bagamoyo and 55% [n = 679] of houses in Ulanga. About half (47% [n = 3157]) of the houses with screened windows in Bagamoyo also had sufficient access to ITNs and in Ulanga, 51% [n = 638] of houses with closed eaves had sufficient access to ITNs (Table 1).

### Household factors associated with houses having window screens and closed eaves in the population

Window screens were more likely to be installed in houses with smaller household size, younger head of household, higher level of education, higher wealth quintile, large houses and sufficient access to ITNs (Table 4). In Ulanga, large house size and higher wealth quintile were associated with window screening (Table 4). Similar to what was observed with window screens, houses that had closed eaves were common among those that head of households were younger people, had high level of education and in the higher wealth quintile in Bagamoyo (Table 5). While in Ulanga, closed eaves were more likely in houses with smaller physical size and sufficient access to ITNs (Table 5).

**Table 4:** Household factors associated with houses that had window screens in the population

Districts	Bagamoyo					Ulanga				
Models	Univariate		Multivariable			Univariate		Multivariable		
Covariates	OR	95% CI	OR	95% CI	P-value	OR	95% CI	OR	95% CI	P-value
Household size										
1 to 5	1		1		0.001	1		1		0.833
6 to 20	0.82	0.74–0.92	0.68	0.59–0.77		1.85	1.28–2.69	1.05	0.67–1.66	
Age group of HH head										
18–24	1		1		0.001	–	–	–	–	–
25–49	0.85	0.70–1.03	0.76	0.61–0.94		–	–	–	–	–
50–99	0.42	0.34–0.51	0.42	0.33–0.53		–	–	–	–	–
Gender of HH head										
Male	1		1		0.409	–	–	–	–	–
Female	0.87	0.77–0.97	1.06	0.92–1.22		–	–	–	–	–
Households with pregnant women										
No	1		1		0.208	1		1		0.123
Yes	1.33	1.03–1.72	1.2	0.90–1.59		1.72	0.82–3.63	2.05	0.85–4.93	
Level of formal education of HH Head										
None	1		1		0.001	–	–	–	–	–
Primary	2.29	2.02–2.61	1.54	1.33–1.79		–	–	–	–	–
Secondary and above	4.81	3.87–6.00	2.1	1.64–2.70		–	–	–	–	–
House size										
Small and average	1		1		0.001	1		1		0.001
Large	1.87	1.69–2.07	2.06	1.82–2.34		5.22	3.66–7.46	2.91	1.86–4.53	
Wealth Quintile										
Lowest	1		1		0.001	1		1		0.001
Low	2.2	1.88–2.56	2.28	1.93–2.68		4.8	1.06–21.70	4.61	1.01–21.06	
Middle	3.66	3.12–4.30	3.33	2.81–3.94		10.07	2.33–43.49	8.92	2.03–39.19	
High	3.33	2.84–3.91	3.18	2.68–3.78		13.43	3.16–57.08	11.3	2.60–49.15	
Highest	9.09	7.55–10.95	7.88	6.44–9.65		55.15	13.38–227.32	41.54	9.67–178.54	
Access to ITNs										
No	1		1		0.001	1		1		0.06
Yes	1.46	1.31–1.62	1.3	1.15–1.47		1.6	0.76–3.36	2.23	0.92–5.40	

The first level in each variable with the value 1 represents the reference group

**Table 5:** Household factors associated with houses that had closed eaves in the population

Districts	Bagamoyo					Ulanga				
Models	Univariate		Multivariable			Univariate		Multivariable		P-value
Covariates	OR	95% CI	OR	95% CI	P-value	OR	95% CI	OR	95% CI	
Household size										
1 to 5	1		1		0.074	1		1		0.711
6 to 20	0.88	0.75–1.03	0.84	0.69–1.02		1.31	1.15–1.49	0.94	0.70–1.28	
Age group of HH head										
18–24	1		1		0.010z	–	–	–	–	–
25–49	1.11	0.87–1.43	1.08	0.81–1.46		–	–	–	–	–
50–99	0.71	0.54–0.92	0.81	0.58–1.12		–	–	–	–	–
Gender of HH head										
Male	1		1		0.817	–	–	–	–	–
Female	0.82	0.69–0.98	0.98	0.80–1.20		–	–	–	–	–
Households with pregnant women										
No	1		1		0.126	1		1		0.928
Yes	0.78	0.53–1.13	0.72	0.48–1.11		0.75	0.42 - 1.34	0.97	0.53 - 1.80	
Level of formal Education of HH Head										
None	1		1		0.001	–	–	–	–	–
Primary	2.37	1.83–3.07	1.55	1.16–2.08		–	–	–	–	–
Secondary and above	8.44	6.32–11.27	2.89	2.06–4.05		–	–	–	–	–
House size										
Small and average	1		1		0.406	1		1		0.001
Large	1.56	1.36–1.80	1.08	0.90–1.30		0.46	0.36–0.60	0.54	0.39–0.73	
Wealth Quintile										
Lowest	1		1		0.001	1		1		0.057
Low	1.56	1.07–2.28	1.46	1.00–2.13		1.38	0.96–1.99	1.62	1.10–2.37	
Middle	1.65	1.14–2.40	1.52	1.05–2.21		1.04	0.72–1.50	1.39	0.93–2.06	
High	2.26	1.59–3.23	1.94	1.35–2.78		0.69	0.48–1.00	1.03	0.69–1.53	
Highest	21.71	15.93–29.59	15.55	11.29–21.44		0.72	0.50–1.04	1.30	0.84–2.00	
Access to ITNs										
No	1		1		0.084	1		1		0.025
Yes	1.91	1.61–2.27	1.19	0.98–1.45		1.76	1.16–2.67	1.72	1.07–2.77	

The first level in each variable with the value 1 represents the reference group

## Discussion

### ITNs coverage in the population

Population access to ITNs and access to ITNs for all sleeping spaces, 3 years following a mass distribution campaign in Bagamoyo was lower than the least 80% coverage targets of the Tanzania National Malaria Strategic Plan. This is consistent with findings that the functional survival time of long-lasting insecticidal nets is less than 3 years in field use [38, 39]. The average interval between campaigns in Tanzania is at least 4 years, thus many people may be unprotected from malaria vectors during the interval.

Family size is an important factor that needs to be considered when planning ITN distribution campaigns according to this study’s finding of low coverage in large households compared to average and small households in both Bagamoyo and Ulanga districts. Larger households may have more children, who are more likely to share sleeping space with their parents or a sleeping space occupied by more than two children. This finding has also been observed in the lake region of Tanzania where household numbers are on average larger, (Ikupa Akim pers. comm). Therefore, our study demonstrates bed net campaigns should consider removing the current limit on the maximum number of nets allowed per household, in order to achieve high ITN coverage among households of large size.

The sleeping pattern, where more than 2 people share a net (crowding) among large households and those with children, has been found to decrease the durability of ITNs due to net stretching, resulting in more rapid loss of ITNs due to damage among this group compared to those with smaller or average household [40], [41], Additional efforts are required to improve population access to ITNs that consider the demography and sleeping pattern of individual households.

Mosquitoes are more attracted to households with an increased number of people [42], thus households with larger family sizes need to have adequate ITNs to impact malaria. ITNs distribution in schools as a keep-up strategy may provide additional coverage for larger households [43]. School-age children tend to have the lowest access to ITNs [44], therefore the school nets program in Tanzania is a useful strategy to help target this vulnerable group.

In Bagamoyo, there was also inequity to access to ITNs among several demographic factors: (1) age groups, especially among households headed by younger adults (18 to 24 years old), perhaps the households with younger heads fall in the reproductive age group and would normally have new children after mass distribution of ITNs; (2) educational attainment of head of households, information on the importance of ITN use for malaria prevention may be lagging among those with less formal education, which has been seen widely, for instance in Ghana [45]; and (3) Wealth status, rich people purchased ITNs when the ones they were given by the campaign became non-functional. On the other hand, in Ulanga, where ITNs were recently distributed, the wealthiest households had lower access to ITNs. This may be attributed to working household members not being present at home during the distribution process, as was observed during the collection of adverse events data at the end of the survey. In a nutshell, it is evident that there was inequity to access to ITNs in the population, as a result, the BCC strategy at the community level may be targeted towards all households to encourage continuous uptake, care of ITNs to improve ITN longevity and to encourage the continued use of damaged ITNs between campaigns, as it has been shown that damaged nets remain insecticidal [46]. It is clear, however, that more effort is needed in improving access to ITNs.

### House modification in the population

A possible explanation for the higher proportion of houses with window screens in Bagamoyo, could be attributed to wealthier households and availability of low-cost screening, being closer to Dar es Salaam, the economic capital of Tanzania [13], as compared to Ulanga, a remote rural area with low income. The majority of the houses in Bagamoyo had opened eaves possibly due to low awareness of closed eaves as a tool to reduce indoor mosquito density [12], and high temperature during the daytime, as found in neighbouring coastal Kenya (Mombasa) [47]. This contrasts with findings in Ulanga where more of the houses blocked their eaves with mud. Having open eaves in houses is an efficient means of indoor cooling [12], which is also a plausible explanation for the observed pattern, especially since Bagamoyo has an average temperature 2 °C higher and greater humidity than Ulanga due to its proximity to the ocean.

The strongest household factor associated with having window screens in both Bagamoyo and Ulanga was wealth. Wealthy households in Bagamoyo were more likely to have closed eaves where window screens were also in common use, while in Ulanga, the poorest households were more likely to close their eaves with mud to prevent mosquito indoor entry [48]. As wealth is strongly associated with window screening, it would be prudent for window screens to receive a subsidy and made more widely available as a long-term vector control intervention to protect all household members while indoor—through indoor mosquito entry barriers. The acceptance of closed eaves in the rural areas do not seem worrisome as it is already a common housing structure among modern houses owned by wealthy people in Tanzania [49]. However, in houses with indoor fires, closing eaves is not advisable as it may increase exposure to biomass particulate matter and induce respiratory illness [50].

In contrast to having access to ITNs for two potential sleepers in Bagamoyo, window screens and closed eaves were more common in households headed by young individuals. This group had more years of formal education and were wealthier than the older age group, as access to education in Tanzania has steadily improved over the last decades [51]. This might have made them more knowledgeable about the importance of the tool in preventing indoor mosquito entry and their wealth afforded them the opportunity to install them in their houses. This also serves as an indicator that younger people are adaptive to the uptake of new tools for controlling malaria. However, installing window screens alone is not sufficient to control malaria [52], because mosquitoes may find their way indoor when doors are left open. The incorporation of insecticides into window screens would improve the efficacy of this tool against mosquitoes as it has improved the protective efficacy of conventional bed nets and other vector control tools [53], providing both individual and community protection—through killing vectors that come into contact with the screens. It is logical that maximizing the concurrent use of multiple vector control tools, i.e. combination of ITNs and insecticidal house screening will assist malaria elimination in Tanzania [54] and also protect against other vector-borne diseases [11], [12]. The combination of ITNs and house screening already occurs, where households with access to one ITN for every two members were more likely to have window screens in Bagamoyo and closed eaves in Ulanga, suggesting that they already understood that using bednets as a stand-alone malaria control tool is not sufficient.

It is important to stress the consideration of household size in vector control delivery system plans. In Bagamoyo, houses with large households were less likely to have access to ITNs and less likely to have window screens, despite the higher possibility of having more children residing in them. The possible reason for this was found, most of the large households were headed by people with low formal education, which reinforces the necessity of BCC strategy incorporation during distribution campaigns.

The study was affected by non-response and desirability bias [55], which might have influenced ITNs access estimates. It was suspected that respondents denied owning at least one ITN in the household due to an expectation of compensation in the form of ITNs after the study. Therefore, it was necessary to use the number of ITNs reported to be used the previous night as a proxy for ownership. This was more reliably measured as interviewers asked the use of each ITN with respect to where it was used in the household. Ideally, surveyors of ITN access should visually inspect the presence of nets in households whenever possible to reduce the possibility of respondents giving misleading answers. The study was unable to establish a standardized comparison between the study areas, as age, sex and educational status of heads of households in the Ulanga district were not collected. Nevertheless, the study recruited a very large number of participants which gave the study strong power to establish associations between the outcomes and covariates.

## Conclusion

This study demonstrates the need to improve access to ITNs and the wider installation of house modification tools for vector control, especially among larger households. Specifically, the study suggests that large family households, households headed by young people, with low formal education, and the poorest households should be specifically targeted in keep-up and universal coverage campaigns, accompanied by BCC strategies for effective maximization of control tools. It is evident in the study that the wider installation of window screens could largely be improved by decreasing the associated costs through government subsidies. Therefore, it is recommended that strategies be put in place to improve access to window screens widely through low-cost screens and insecticidal screens to maximize their use in combination with ITNs to further control malaria as well as other vector-borne diseases.

## Data Availability

Not applicable.

## Abbreviations

ANC: Antenatal Care
BCC: Behaviour Change Communication
DHS: Demographic Health Survey (Tanzania)
GIS: Geographical Information System
IHI: Ifakara Health Institute
ITNs: Insecticide Treated Nets
NMCP: National Malaria Control Programme
NMSP: National Malaria Strategic Plan
PCA: Principal Component Analysis
SES: Socioeconomic Status
UCC: Universal Coverage Campaign
WHO: World Health Organization
